# Effect of compression pre-force and web openings on torsional strength of UHPC hollow beams using numerical and mathematical modeling

**DOI:** 10.1038/s41598-025-10834-0

**Published:** 2025-07-16

**Authors:** Ahmed M. El-Basiouny, Hamed S. Askar, Mohamed E. El-Zoughiby

**Affiliations:** Structural Engineering Department, Faculty of Engineering, Mansoura University, Mansoura, Egypt

**Keywords:** Ultra-high-performance concrete, Torsional strength, Compression pre-force, Web openings, Numerical study, Finite element

## Abstract

In this paper, a new formula is proposed to accurately predict the torsional strength of ultra-high-performance concrete (*UHPC*) solid and hollow beams with and without openings. A comprehensive parametric numerical study is carried out to investigate the effect of both compression pre-force and web openings parameters on the torsional strength of *UHPC* solid and hollow beams. This study is based on numerical results of 44 applied finite element (*FE*) models with Abaqus software. Furthermore, five recent available formulas, capable of predicting the torsional strength of *UHPC* beams, were introduced and compared and to clarify their deficiency, three of them were calibrated to the 44 *FE* models. The proposed new formula was not only compensated for the deficiency of former formulas, but it also included parameters not previously considered. To examine its validity, the results of the derived formula were reviewed with those of 44 *FE* models and other 46 previously tested *UHPC* beams. In both cases, the proposed new formula demonstrated high accuracy in predicting the torsional strength of *UHPC* beams.

## Introduction

Due to its extraordinary mechanical properties and durability, ultra-high-performance concrete (*UHPC*) is a specialized class of concrete that is increasingly used in various applications, especially structures subjected to extreme stresses and environmental conditions^1–4^. Enhanced compressive strength (in excess of 150MPa, under thermal curing^5^, high tensile, flexural and shear strengths^6^, very high fracture, toughness and ductility^7^, exceptional endurance and longevity, etc., make *UHPC* a superior choice that frequently used in infrastructure projects, particularly in bridge construction^8,9^. Zhou et al.^10^ presented a list of bridges around the world that partially or completely constructed using *UHPC*.

Torsion in skew-curved concrete bridges is a matter of concern^11^. To effectively resist torsional stresses, *UHPC* appears as a practical solution. In addition, applying a longitudinal compression pre-force can help to counteract such stresses.

Recently, importance of creating transverse openings in web of concrete bridges has grown. Openings are not only used to pass installation, but also to simplify maintenance of bridges, decrease the self-weight of long-span bridges, maintain the air quality in enclosed environment structures and enhance its sustainability. Moreover, architecturally, the presence of openings contributes to the creation of unique designs with aesthetic appeal.

Although both compression pre-force and openings can greatly affect the torsional behavior of *UHPC* elements, recent literature has not given sufficient attention to either.

First trials for predicting the torsional capacity of *UHPC* were based on traditional theories, developed for normal strength concrete; Hossain et al.^12^ and Hsu et al.^13^. Such theories supposed that concrete torsional strength is completely governed by stirrups, thereby disregarding the substantial contribution of steel fibers and the high tensile strength of *UHPC*. Therefore, application those assumptions to *UHPC* resulted in illogical results. This urged some researchers to examine the influence of steel fibers on the torsional capacity of *UHPC* beams. Based on an experimental work in 2012, Empelmann and Oettel^14^ found that the addition of steel fibers significantly improved the torsional capacity of *UHPC* box girders.

Later, Yang et al.^11^, Fehling et al.^15^ and Kwahk et al.^16^ conducted experimental studies on *UHPC* beams to examine the contribution of steel fibers, longitudinal and transverse steel on their torsional behavior. Thereafter, mathematical models capable of calculating the torsional strength of *UHPC* beams were presented by Fehling^15^ and Kwahk^16^. The strategy of these models was intended to estimate the torsional strength of *UHPC* beams by combining the contribution of transverse steel and steel fibers. Nevertheless, they did not consider the potential effect of fibers dimensions and volumetric ratio or type of beam cross-section (solid or hollow) on the torsional capacity.

Another trial was implemented in 2022 by Mitobaba et al.^17^ to develop an analytical model capable of predicting the torsional capacity of *UHPC* beams. The model considered the effect of type of cross-section and the contribution of transverse steel and steel fibers, but ignored the probable effect of the number of longitudinal reinforcing steel-bars. Compensating for this deficit, in 2023, Cao et al.^18^ proposed a new formula that considered the contribution of longitudinal and transverse steel and the tensile strength of *UHPC*. On the other hand, the type of beam cross-section and characteristics of steel fibers (volumetric ratio and dimensions) were not considered.

## Research significance

The aim of the current comprehensive numerical investigation is to develop a new model for accurately predicting the torsional strength of *UHPC* solid and hollow beams. The proposed model addresses several limitations identified in previous models by enhancing predictive accuracy and integrating additional parameters into a single unified expression, rather than treating them separately. These parameters include the presence of (square, rectangular or circular) web openings with varying sizes and numbers, as well as effect of compression pre-force. Accordingly, the study involved evaluating the accuracy of existing models, highlighting their deficiencies and limited comprehensiveness and proposing improvements that incorporate previously unaccounted parameters.

## Numerical parametric study

In order to specify the main parameters influencing on the torsional strength of *UHPC* beams, 44 *UHPC* beams were modeled using *FE* method with Abaqus software. All input data would be presented in the following sections.

### Finite element parameters

To accurately represent concrete, a brittle material, with its cracking and crushing behavior in Abaqus software^19^, the concrete damage plasticity (*CDP*) model is the most suitable one^20^. The presence of fibers and fine-particles in *UHPC* makes its tension, compression and shear behaviors differ from conventional concrete^20^. In the current study, all differences were considered based on recent related researches^20–24^. Table 1 presents some prerequisites of defining the *CDP* model in Abaqus software.

**Table 1:** *CDP* parameters, Fakeh et al.^20^.

Dilation angle (Ψ)	55
Eccentricity	0.10
Stress ratio f_b0_/f_c0_	3
Viscosity parameter	0.001

As illustrated in Table 1, unlike the previous studies, the dilation angle and the stress ratio were considered 55 and 3, respectively. These values were considered based on Fakeh et al.^20^ recommendations, where multiple axial compression tests concluding the most accurate inputs for *UHPC* were presented.

The stress–strain curve for *UHPC* concrete in compression, defined in *CDP* model, consists of ascending and descending parts. Based on several available models (Yan^21^, Zhao et al.^22^, Wang et al.^23^ and Graybeal^24^), it was concluded by Fakeh et al.^20^ that the best simulating model for the stress–strain curve for *UHPC* is the one proposed by Graybeal^24^ for the ascending part and by Prem et al.^25^ for the descending part. The *UHPC* elastic modulus ${E}_{c}$ was calculated utilizing the formula presented by Grybeal^24^.

In Abaqus, the torque lever arm was assumed rigid and complete bond was assumed between the concrete and reinforcing steel bars embedded in it.

For the considered *FE* models, the cube compressive and tensile strengths of *UHPC* were 120MPa and 7MPa, respectively. The tensile stress–strain curve for steel has been used based on Zhu^26^ recommendations. Furthermore, the longitudinal and transverse reinforcement yield stresses have been taken as 350 MPa and 240 MPa, respectively.

In order to determine the proper mesh size, two previously tested *UHPC* beams subjected to torque were modeled with Abaqus software: Beam SH-P0-F1.5-L1-S1(D13) tested by Kwahk et al.^16^ and Beam UPF1(0.9)28 tested by Fehling et al.^15^. For each beam, five mesh sizes have been examined: 300, 200, 120, 60 and 40 mm. The torsional strength corresponding to each mesh size was compared with the experimental results, Table 2. Obviously, as Table 2 illustrated, the appropriate mesh size is 60 mm.

**Table 2:** Experimental and *FE* results for different mesh sizes.

Refs.	Torsional capacity, kN-m					
	*FE*					Exp
	Mesh size, mm					
	40	60	120	200	300	
^16^	101.2	101.4	102	103.4	103.4	100.7
^15^	44.6	44.65	47	57	57.3	46.32

### Verification

**Fig. 1:**
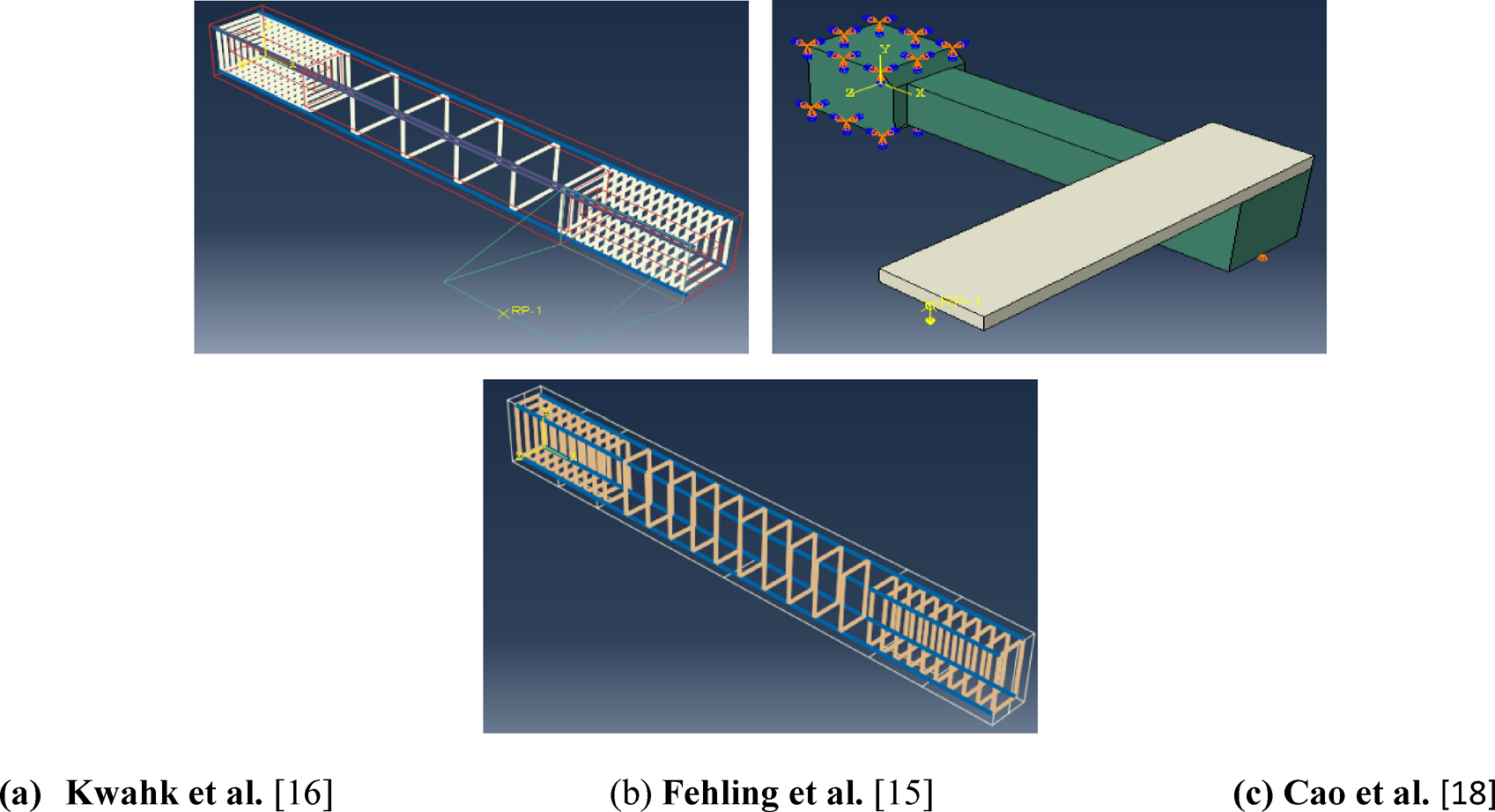
Finite element models for previous studies using Abaqus software.

**Fig. 2:**
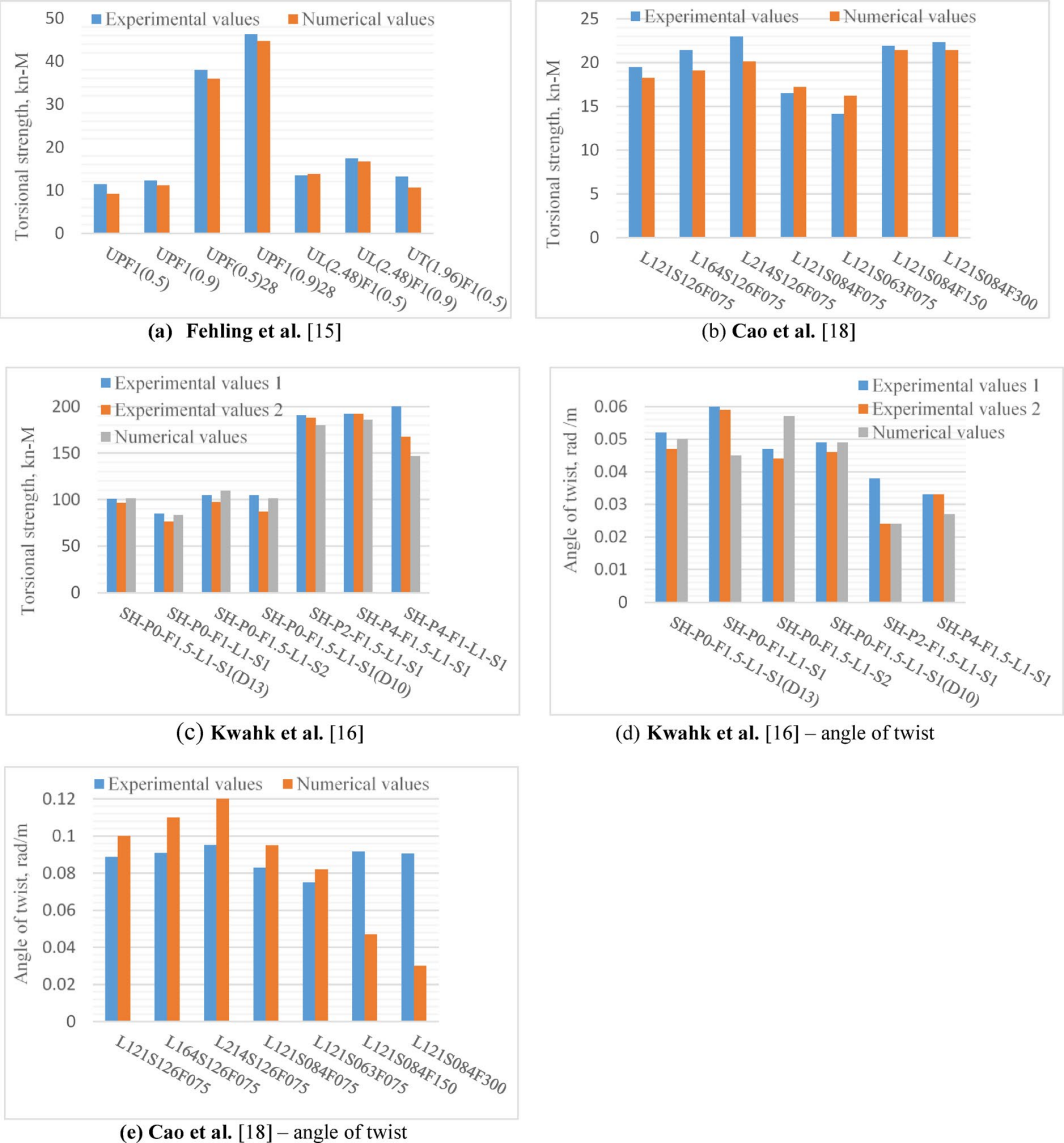
Comparison between numerical and experimental torsional strength.

To evaluate the *FE* results, 21 tested *UHPC* beams from previous related studies^15,16,18^ were modeled using Abaqus software. The studied parameters included longitudinal steel, stirrups, section type and prestressing force, Fig. 1. The obtained numerical results were compared to the experimental results; ultimate torsional capacity and angle of twist ${T}_{u}$ and ${\theta }_{u}$, respectively, Fig. 2.

Figure 2 indicated an acceptable agreement between the numerical and experimental results from references^15,16^ and^18^ with mean values 91.7%, 102% and 97.4%, respectively, for the ratio of the ratio ${T}_{u}^{FE}$/${T}_{u}^{Exp.}$. Also, the values of ${\theta }_{u}^{Exp.}$ and ${\theta }_{u}^{FE}$ were very close. In Kwahk et al.^16^ study two identical specimens were fabricated for each beam type. Therefore, each beam type had two corresponding torsional strength values.

### Geometry of *UHPC* beam models

**Fig. 3:**
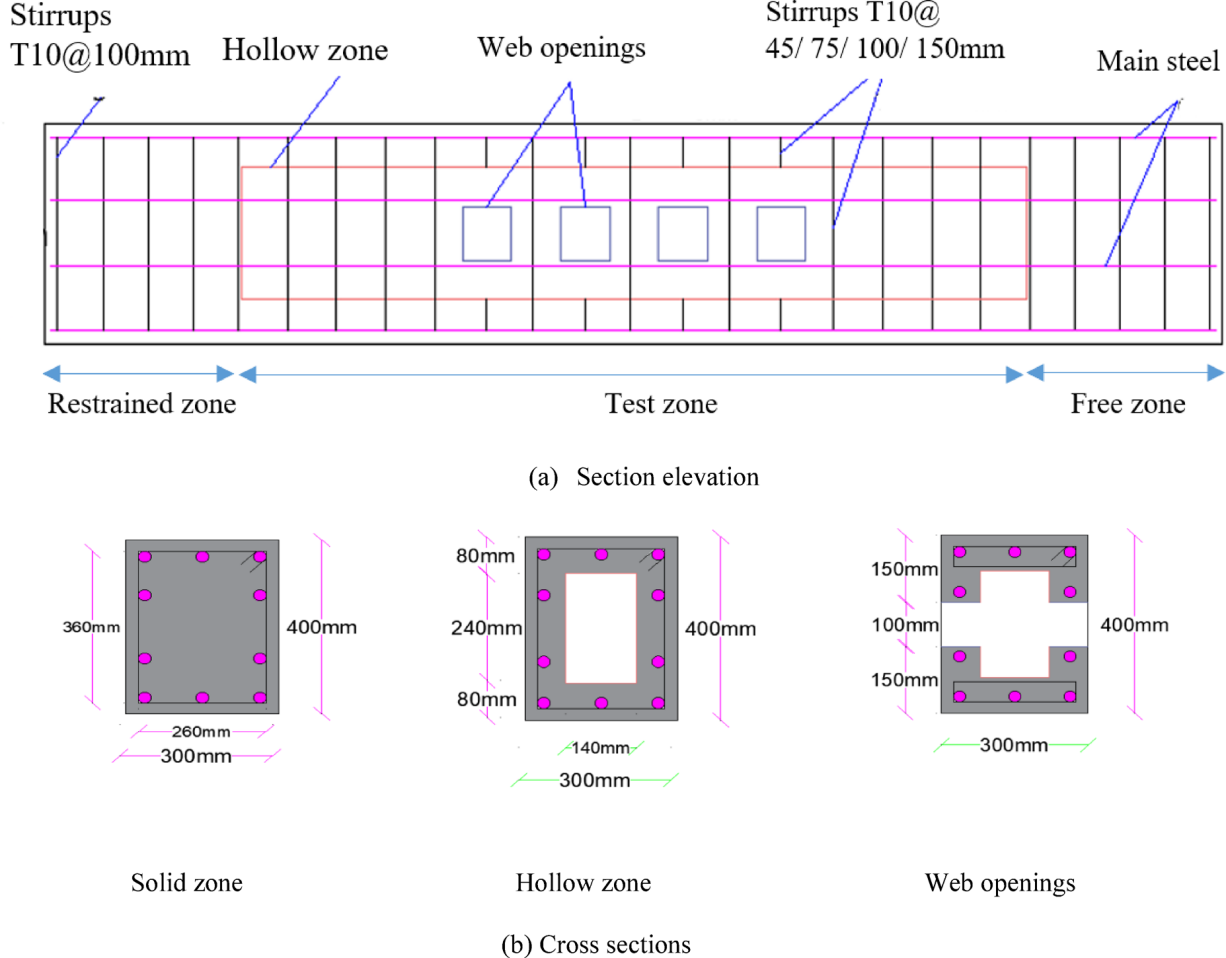
Details of the finite element models.

Each beam has length of 2400mm, width of 300mm and height of 400mm. The torque lever arm is 650mm away from the axis of rotation passing through the centroid of beam section. Each beam is fixed at one end and free at the other end. Both ends, each of 400mm length, are modeled as solid zones, where the spacing of stirrups is 100mm. At one solid end, boundary conditions are defined to represent a fixed support, while at the other solid end, they are defined to simulate a roller support. It allows beam rotation and prevent formation of any additional load effects other than torque moment, Fig. 3.

### Study parameters

Table 3 presents 16 *UHPC* hollow beams and 1 solid beam classified into 5 groups: *A, B, C, D*, and *E*. In all groups, the diameter of the transverse reinforcement is 10mm and the number of longitudinal reinforcing bars is 8 bars. For the first 4 groups, the thickness of hollow section *t* is 80mm and the distance between stirrups *s* is unified in each group but differs from one group to another. Still with the first four groups and based on 5 recent investigations^11,15,16,18^ and^27^, three longitudinal reinforcement ratios are selected and assigned one for each of the three beams in each group. The last group consists of five beams having similar longitudinal and transverse reinforcement ratios. One beam is solid and the others differ in thickness of hollow section that is; 60 mm, 80 mm, 100 mm and 120 mm.

**Table 3:** Classification and torsional strength of *UHPC* beams.

Group	Beam	Longitudinal reinforcement		Transverse reinforcement		Thickness, *t,* mm	Torsional strength *T*_*u*_, kN-m
		content	ratio ${\rho }_{l}$^11^	*s*, mm	${\rho }_{t}$^11^		
A	A1	8*T*10	0.0073	150	0.007	80	104.2
	A2	8*T*12	0.010				110
	A3	6*T*12 + 2*T*16	0.0125				113.6
B	B1	8*T*10	0.0073	100	0.011	80	110.5
	B2	8*T*12	0.010				117
	B3	6*T*12 + 2*T*16	0.0125				120.7
C	C1	8*T*10	0.0073	70	0.016	80	116
	C2	8*T*12	0.010				123.9
	C3	6*T*12 + 2*T*16	0.0125				126.7
D	D1	8*T*10	0.0073	45	0.025	80	122.5
	D2	8*T*12	0.010				126
	D3	6*T*12 + 2*T*16	0.0125				127.7
E	E1	8*T*12	0.010	100	0.011	60	104.3
	E2	8*T*12	0.010			80	117
	E3	8*T*12	0.010			100	127.6
	E4	8*T*12	0.010			120	131
	E5	8*T*12	0.010			Solid	130

To simulate compression pre-force, 10 *UHPC* beams, from the five groups, were subjected to an axial compression pre-force (*A*1, *A*2, *A*3, *B*2, *B*3, *C*3, *D*3, *E*1, *E*2 and *E*3). The values of applied force were; 100 kN, 200 kN and 300 kN, Table 4.

**Table 4:** Effect of compression pre-force on torsional strength of *UHPC* beams.

Study parameters	Beam	${T}_{u}$ kN-m	Compression pre-force, kN	${T}_{u}^{c}$, kN-m	$\frac{{T}_{u}^{c}}{{T}_{u}}$
Longitudinal reinforcement	A1-200	110.5	200	122.5	1.11
	A2-200	117	200	126	1.08
	A3-200	120.7	200	128.2	1.06
Transverse reinforcement	D3-200	127.7	200	147.1	1.15
	C3-200	126.75	200	137.15	1.08
	B3-200	120.7	200	128.2	1.07
	A3-200	113.6	200	120.8	1.06
Thickness	E1-200	104.3	200	113.25	1.09
	E2-200	117	200	126	1.08
	E3-200	125	200	134.9	1.08
Compression pre-force	B2-100	117	100	122	1.04
	B2-200		200	126	1.08
	B2-300		300	129	1.105
	D3-100	127.7	100	144	1.13
	D3-200		200	147.9	1.15
	D3-300		300	149.5	1.17
	A1-100	104.2	100	103.4	1.00
	A1-200		200	113.2	1.09
	A1-300		300	118.8	1.14

Eventually, the two beams *B*1 and *B*3 were utilized to investigate the effect of creating squared symmetric web openings. The opening side length was either 100mm or 200mm and the number of openings was 2, or 4, or 6, or 8, Table 5.

**Table 5:** Effect of web openings on torsional strength of *UHPC* beams.

Beam	Opening		$\frac{{T}_{u}^{o}}{{T}_{u}}$
	Dim., mm	Nos	
B1	100⨯100	2	0.90
		4	0.85
		6	0.77
		8	0.69
	200⨯200	2	0.80
		4	0.52
B3	100⨯100	2	0.90
		4	0.79
		6	0.70
		8	0.68
	200⨯200	2	0.80
		4	0.52

**Fig. 4:**
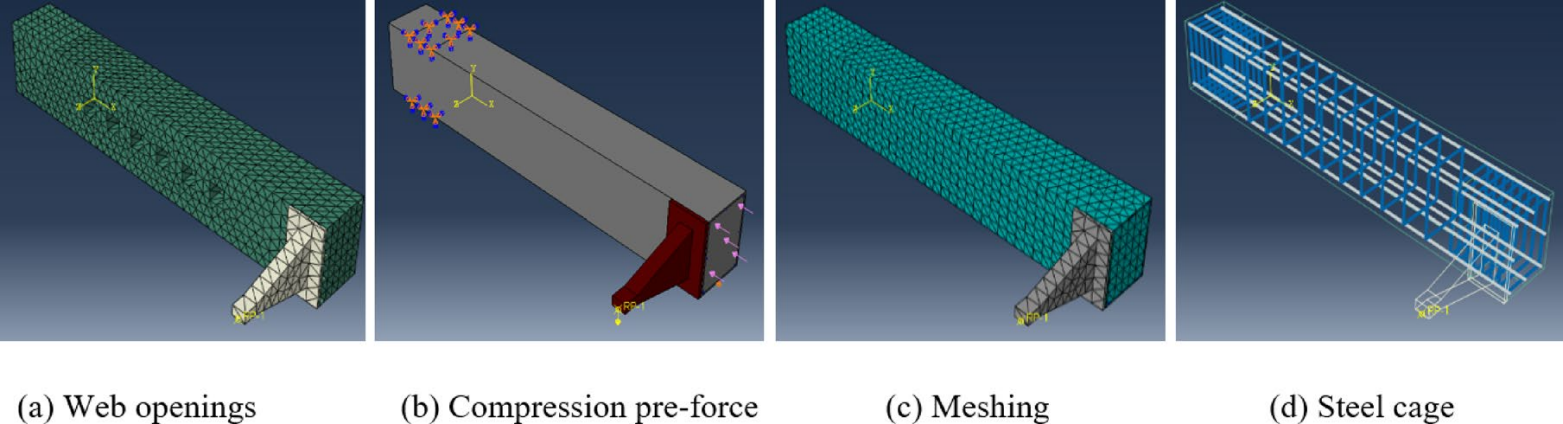
Finite element models based on Abaqus software.

Figures 3 and 4 show some details of *FE* models with and without web openings. Tables 4, 5, and 6 present the resulted torsional strength of the modeled *UHPC* beams. In which ${T}_{u}^{c}$ and ${T}_{u}^{o}$ mean torsional strength of pre-compressed *UHPC* beams and *UHPC* beams with openings, respectively.

**Table 6:** Applying the previous formulas to the *FE* models.

Group	Beam	${T}_{u}^{FE}$, kN-m	*T*_*u*_^18^, kN-m	*T*_*u*_^17^, kN-m	*T*_*u*_^16^, kN-m	$\frac{{T}_{u}}{{T}_{u}^{FE}}$^18^	$\frac{{T}_{u}}{{T}_{u}^{FE}}$^17^
A	A1	104.2	85.211	87.7	105.4	0.82	0.84
	A2	110	102.32	105.7		0.93	0.96
	A3	113.6	111.78	115.7		0.98	1.018
B	B1	110.5	82.75	90.77	118.7	0.75	0.82
	B2	117	99.3	110		0.85	0.94
	B3	120.7	108.56	120.7		0.90	1.00
C	C1	116	83.7	94.3	135.7	0.72	0.81
	C2	123.9	100.2	115		0.81	0.93
	C3	126.7	110.4	126.7		0.87	1.00
D	D1	122.5	88	100	141.9	0.72	0.82
	D2	126	105.6	123.6		0.84	0.98
	D3	127.7	115.37	136.6		0.90	1.07
E	E1	104.3	99.3	102.9	114.6	0.95	0.99
	E2	117	99.3	110	118.7	0.85	0.94
	E3	127.6	99.3	114.4	118.9	0.78	0.92
	E4	131	99.3	116.87		0.76	0.89
	Mean^18^					0.84	
	Mean^17^						0.93

## Numerical results and discussion

**Fig. 5:**
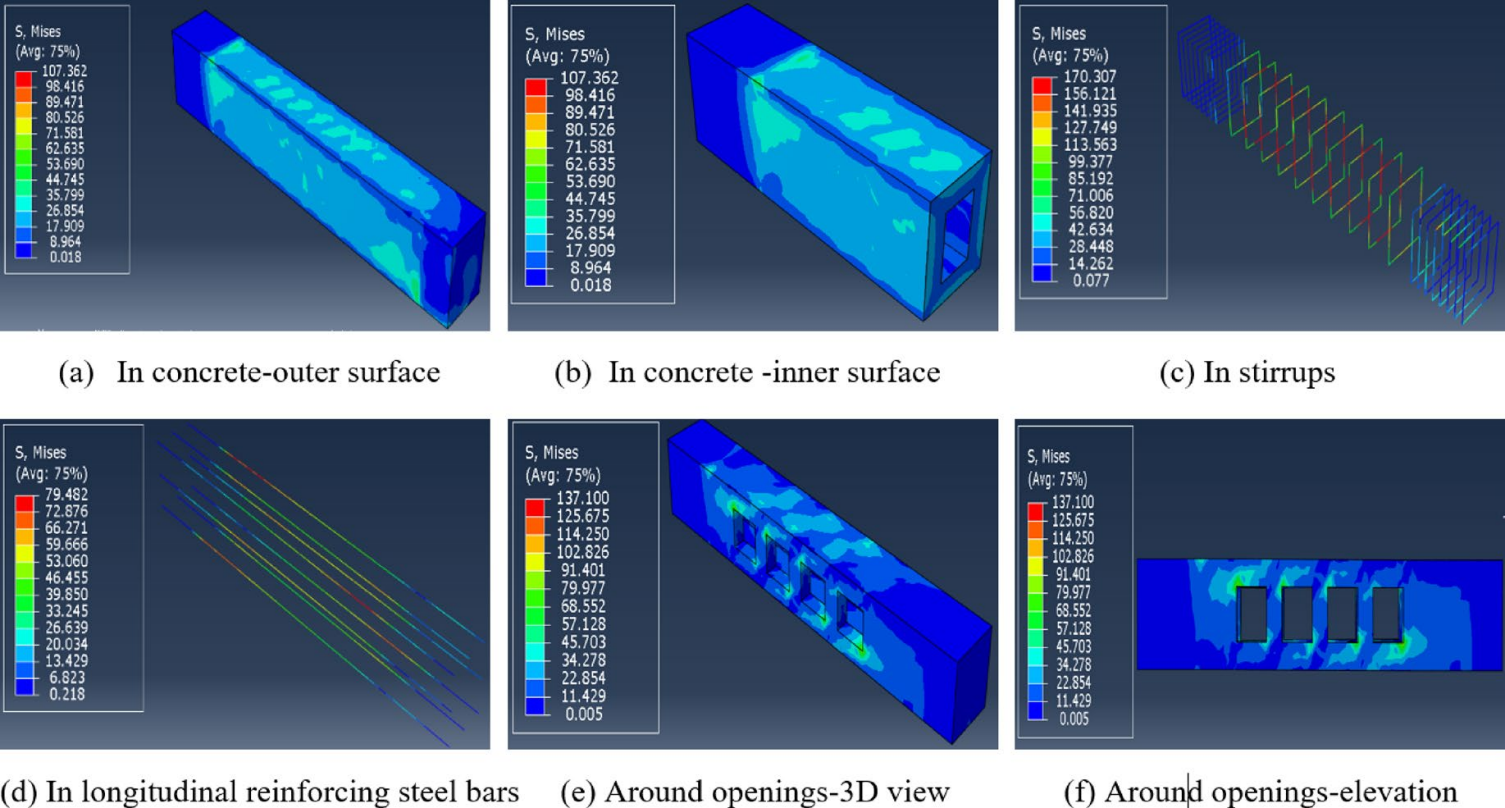
*FE* models using Abaqus software. (**a**) torsional stresses at outer surface of concrete. (**b**) observed torsional stresses at inner surface of concrete. (**c**) values of torsional stresses in the stirrups. (**d**) values of torsional stresses in the longitudinal reinforcing steel bars. (**e**) stress concentration around the openings in isometric view. (**f**) stress concentration around the openings in section elevation.

Some selected *FE* results are next displayed and discussed. Figure 5a, b illustrate the noticed torsional stresses at outer and inner surfaces of concrete, respectively. Figure 5c shows higher values of torsional stresses in the stirrups in the test hollow zone than those existing in the two solid restrained and free zones. Furthermore, the middle third of the longitudinal reinforcing steel bars was exposed to higher torsional stresses than those noticed at ends, Fig. 5d. Figure 5e, f show a stress concentration around the openings.

**Fig. 6:**
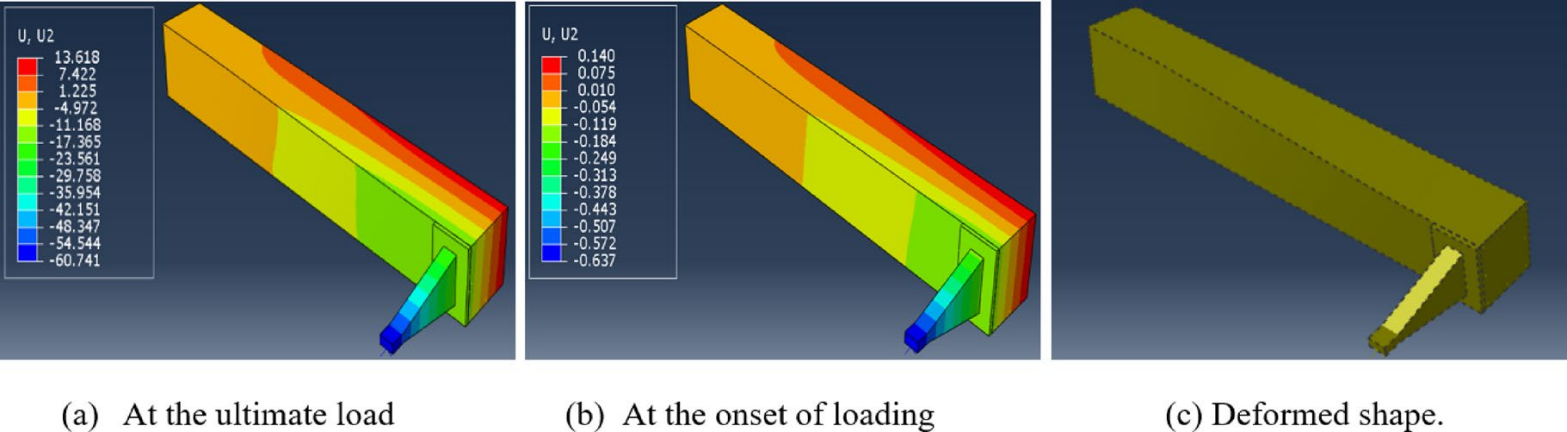
Vertical displacement for *UHPC* beams. (**a**) vertical displacement for *UHPC* beams at the ultimate load. (**b**) vertical displacement for UHPC beams at the beginning of loading. (**c**) deformed shape of one tested beam.

The vertical displacement of the *FE* models shown in Fig. 6a, b had its max. negative value at the edge of the lever arm where the load was applied. Also, it showed positive values at the upper surface of tested beam and negative values at its front surface. The vertical displacement values observed in Fig. 6a, at the ultimate load, were higher than ten-times its corresponding value at the beginning of loading, Fig. 6b. Figure 6c shows the deformed shape in one model that is a natural and expected.

### Influencing parameters affecting *UHPC* beams

As previously mentioned in Section “Introduction”, the contribution of longitudinal and transverse reinforcing steel and fibers to the torsional capacity of *UHPC* beams was previously considered in literature, and therefore it would be briefly discussed. The next part would focus on other influencing parameters affecting the torsional strength of *UHPC* beams, such as compression pre-force, web openings and beam thickness.

#### Longitudinal and transverse reinforcement ratios

As illustrated in Table 3, increasing the ratio of longitudinal reinforcement ${{\varvec{\rho}}}_{{\varvec{l}}}$ from 0.0073 to 0.01 had a considerable effect on the torsional strength of *UHPC* beams. For this, beams; *A*2, *B*2, *C*2 and *D*2 showed higher torsional strength than that of beams; *A*1, *B*1, *C*1 and *D*1, respectively. However, this effect was noticed to be slightly weakened, when ${\rho }_{l}$ had increased from 0.01 to 0.0127, in beams; *A*3, *B*3, *C*3 and *D*3.

On the other side, the larger the ratio of transverse reinforcement ${\rho }_{t}$ the larger the torsional strength of the tested *UHPC* beams.

#### Thickness

Table 3, considering the beams of Group *E*, presents the effect of changing the thickness of the *UHPC* hollow beams on their torsional strength. Increasing the thickness from 60 mm (0.20 of beam width and 0.15 of its height), as in Beam *E*1 to 80 mm (0.27 of beam width and 0.20 of its height), as in Beam *E*2 and to 100 mm (0.33 of beam width and 0.25 of its height), as in Beam *E*3, increased the torsional strength by 13% and 23%, respectively. But the increase in the torsional strength was not clearly observed when the thickness increased to 120 mm (0.40 of beam width and 0.30 of its height), as in Beam *E*4, or even in the solid Beam *E*5 showing approximately the same torsional strength of that of Beam *E*4. This can be attributed to thin walled tube theory.

#### Compression pre-force

As noticed in Table 4, applying a compression pre-force to the examined *UHPC* beams, enhanced their torsional strength. However, increasing the content of longitudinal reinforcement, reduced the effect of applying the compression pre-force. For example, for Group *A*, the increase in the torsional strength of Beam *A*1, due to applying compression pre-force of 200kN was 11%. Despite the same applied pre-force, the increase was greater than that of Beams *A*2 and *A*3; 8% and 6%, respectively.

In contrast, when applying a compression pre-force, a direct proportionality was observed between the transverse reinforcement content and the enhancement of the torsional strength of the beams. For instance, the increase in the torsional strength of Beam *D*3 was 15%, while it decreased to 8%, 7% and 6% for Beams *C*3, *B*3 and *A*3, respectively, although all the beams were subjected to the same compressive pre-force of 200kN.

The enhancement in the torsional strength of the beams in Group *E*, for applying compression pre-force of 200kN, was not affected by the varying the thickness of the beams. As the torsional strength of these beams increased approximately by the same ratio.

Eventually, among beams *A*1, *B*2 and *D*3, applying compression pre-force of 100kN to Beam *D*3, led to a noticeable enhancement in its torsional strength by 13%. The observed increase surpassed that of Beam *B*2 and was nearly equivalent to the improvement exhibited by Beam *A*1, those were precompressed by 30 kN. This can be attributed to the high value of ${\rho }_{t}$ for Beam *D*3; 0.025, that magnified the effect of compressive pre-force.

#### Web openings

As observed in Table 5, creating two squared web openings with a side length of 100mm in beams *B*1 and *B*3 led to a loss in their torsional strength by only 10%, compared to that without openings. Also, increasing the number of web openings to 4, 6 and 8, in the two beams, led to further reduction in their torsional strength by average ratio of 17.5%, 26.5% and 31.5%, respectively.

The loss in the torsional strength of the modeled beams was observed to be increased to 48%, after creating 4 squared openings each with a side length of 200mm. This loss exceeded that resulted from creating 8 squared openings each with a side length of 100mm.

## Torsional strength prediction

This section provides a comprehensive comparison between five recent formulas, devoted to predict torsional strength of *UHPC* beams based on thin-walled tube theory^28^ and modified space truss analogy^29^. Applying these formulas to the studied 44 *FE* models helps to examine the formulas accuracy and comprehensiveness.

### Existing formulas

Firstly, five formulas previously developed by Fehling et al.^15^, Kwahk et al.^16^, K-UHPC Technology^30^, Mitobaba et al.^17^ and Cao et al.^18^, are next presented. The first three formulas^15,16,30^ have almost the same parameters, but the second formula additionally considers the prestressing force. Therefore, this formula and the last two formulas^17,18^ are applied to the results of *FE* models. The comparison of results among the three formulas are presented in Table 6. However, the five formulas are next displayed and discussed in detail.

The first mathematical model proposed by Fehling et al.^15^ in 2013, is as follows:

1 $$T_{u} = \frac{{2A_{0} A_{t} f_{yv} }}{s }\cot \,\theta + \frac{{2A_{0} A_{w} \sigma_{cfo} }}{{\cos \theta \times \left( {h - t_{eff} } \right)}}$$

where *T*_*u*_ is the torsional strength of *UHPC* beams, ${A}_{0}$ is the area enclosed by shear flow, ${A}_{t}$ is the cross-sectional area of one leg of a stirrup, ${f}_{yv}$ is the yield stress of stirrups, $s$ is spacing of stirrups, $\theta$ is the angle of inclined cracks, $h$ is the beam height, ${t}_{eff}$ is the beam effective thickness, ${\sigma }_{cfo}$ is the tensile strength of *UHPC* beam after cracking, $s$ is the distance between stirrups and ${A}_{w}= {t}_{eff}\times \left(h-{t}_{eff}\right)/sin \theta$.

Equation (1) has taken into account concrete tensile strength and stirrups characteristics (spacing, yield stress and diameter). But, fibers properties (length, diameter and volume fraction), longitudinal steel, compression pre-force, type of concrete section and existing of web openings are not considered.

The second model proposed by Kwahk et al.^16^ in 2015, is given by:

2 $$T_{n} = \frac{{2A_{0} A_{t} f_{yy} }}{s}\cot \,\theta + 2A_{o} f_{t} t {\text{cot}}\,\theta$$

where *T*_*n*_ is the torsional strength of *UHPC* beams*,* ${f}_{yy}$ is the yield stress of stirrups, *t* is the effective thickness and ${f}_{t}$ is the tensile strength of *UHPC*.

In contrast to Eqs. (1), (2) has taken into account the effect of prestressing force and ignored effect of the diameter of the longitudinal steel reinforcing bars. Therefore, the beams in the same group of *A, B, C* and *D* showed the same torsional strength, as a result of applying Eq. (2), Table 7. Furthermore, the model has not considered the effect of web openings, type of concrete section and fibers properties.

**Table 7:** differences between the existing models.

Variable		Fehling^15^	Kwahk^16^	K-UHPC^30^	Mitobaba^17^	Cao^18^	Current model
Longitudinal steel	Yield stress and diameter	×	×	×	✓	✓	✓
	ratio	×	×	×	×	✓	✓
Transverse steel	Yield stress, diameter and spacing	✓	✓	✓	✓	✓	✓
Concrete tensile strength		×	✓	✓	✓	✓	✓
Fiber characteristics (dimensions and ratio)		×	×	×	✓	×	✓
Section type (solid or hollow)		×	×	×	✓	×	✓
Torsional plastic resistance moment for section		×	×	×	✓	×	✓
Inclination angle of cracks		✓	✓	✓	×	✓	✓
Concrete tensile strength after cracking		✓	×	×	×	×	×
Compression pre-force		×	✓	×	×	×	✓
Web openings		×	×	×	×	×	✓

The third model developed by K-UHPC Technology^30^ in 2018, is as follows:

3 $$T_{d} = \upphi \frac{{2A_{0} A_{v} f_{yvd} }}{s}cot \,\theta + 2\,\upphi \,A_{o} \,f_{td } t\,\cot \,\theta$$

where ${T}_{d}$ is the torsional strength of *UHPC* beams, ${A}_{v}$ is the cross-sectional area of one leg of a stirrup, ${f}_{yvd}$ is the yield stress of stirrups, $\upphi$ is constant value of 0.75 and ${f}_{td}$ is the tensile strength of *UHPC*.

Also, as in Eqs. (1), (3) has not taken into consideration fibers characteristics, compression pre-force, type of concrete section and existence of web openings.

The fourth mode developed by Mitobaba et al.^17^ in 2022, is as follows:

4 $$T_{u} = f_{ut} V_{f } \lambda_{f} W + 0.9 \sqrt {\zeta_{Ru} } \frac{{f_{yv} A_{st1} }}{s} A_{cor} { }$$

where ${T}_{u}$ refers to the torsional strength of *UHPC* beams, ${f}_{ut}$ is the *UHPC* tensile strength, ${V}_{f}$ is the fibers volume of fraction,${\lambda }_{f}$ is the length of one fiber divided by its diameter, ${A}_{cor}$ is the area enclosed by shear flow, ${A}_{st1}$ is the cross-sectional area of one leg of a stirrup and $W$ is the resisting plastic moment of the cross-section. It is given by $\frac{\left(3h-b\right) \times {b}^{2}}{6}$ for solid section and $\frac{\left(3h-b\right) \times {b}^{2}}{6}-\frac{{\left(b-2t\right)}^{2}}{6} (3\left(h-2t\right)-(b-2t)$ for hollow section where $b$, $h$ and $t$ are *UHPC* beam width, height and thickness, respectively. ${\zeta }_{RU}$ is the reinforced fiber strength ratio that quals $(1+{\zeta }_{ut })\times ({\zeta }_{ut}+\zeta )$ where $\zeta$ is the reinforcement bars strength ratio and ${\zeta }_{ut}$ is the stirrup strength ratio. $\zeta =\frac{S {A}_{st1} {f}_{y}}{{f}_{yv} {A}_{svl} {U}_{cor}}$ and ${\zeta }_{ut}= \frac{S t {f}_{ut}}{{f}_{yv} {A}_{svl}}$. In which ${A}_{st1}$ is the cross-sectional area of one longitudinal steel bar, ${f}_{y}$ is the yield stress of longitudinal steel bars, ${U}_{cor}$ is the interference of shear flow and ${A}_{svl}$ is the cross-sectional area of one leg of a stirrup.

Applying of Eq. (4) to the *FE* models has proven its high accuracy in predicting the torsional strength of *UHPC* beams with mean value of 93%, Table 6. Equation (4) included detailed data regarding stirrups, fibers, section type and concrete characteristics, but it has not considered number of longitudinal reinforcing steel bars. Consequently, applying this formula to two identical *UHPC* beams but differ in number of longitudinal steel reinforcing bars will produce similar results. Also, web openings and compression pre-force have not considered.

The last model proposed by Cao et al.^18^ in 2023, is as follows:

5 $$T_{n} = 2\sqrt \zeta \frac{{A_{cor} f_{yv } A_{sv1} }}{s} + 2\alpha \,A_{cor} \,f_{t} t\,\cot \,\theta$$

where $\zeta$ is the reinforcement strength ratio of longitudinal reinforcement and stirrup of torsion member that equals $\frac{S {A}_{sl} {f}_{yl}}{{f}_{yv} {A}_{sv1} {U}_{cor}}$, ${A}_{cor}$ is the area enclosed by inner surface of stirrups, ${A}_{sv1}$ is the cross-sectional area of one leg of a stirrup, $\alpha$ is a correction factor equals 0.5, ${f}_{yl}$ is the yield stress of longitudinal steel bars and ${A}_{sl}$ is the total cross-sectional area of longitudinal reinforcing steel bars.

Though Eq. (5) compensated for some deficits of the first 4 formulas, it has ignored the type of concrete section and fibers characteristics which considered in Eq. (4)^17^. Furthermore, applying Eq. (5) to the *FE* models, as in Table 6, led to un realistic results; each beam in Group *D* showed higher torsional strength than its corresponding in groups *A* and *B.* Although, distance between stirrups in the beams in group *D* is longer than that in groups *A* and *B*. Table 7 highlights the conceptual differences between the existing models and the developed model proposed in the current study (Eq. 15). Furthermore, Table 8 presents a qualitative comparison that clearly outlines the physical assumptions underlying each of the previous models.

**Table 8:** Physical assumptions underlying each of the previous models.

Study	Adopted theory	Physical assumptions
Kwahk^16^	The thin-walled tube	1. The thin-walled tube theory was used in order to take into account the contribution of the tensile strength of *UHPC*, provided by the steel fibers, to the torsional strength of the beam 2. The inclination angle of cracks was derived using the Mohr circle and subsequently validated through experimental results 3. The study recommended that design should exploit effectively the contribution of the steel fiber in *UHPC* structures subjected to torsion
Fehling^15^	The thin-walled tube	1. Before cracking concrete was considered isotropic material and the reinforcement did not take part in beam torsion strength 2. After cracking concrete is no longer isotropic and the contribution of the reinforcement increases gradually 3. The Euro code^31^ definition of the effective thickness of the tube wall was sufficient to calculate the contribution of steel fibers and either longitudinal or transverse steel to the torsional strength of *UHPC* beams
Mitobaba^17^	The space truss analogy and Chinese code^32^ specifications	1. The model accounted the plastic ultimate torsional strength of *UHPC* 2. The beam was considered a thin tube resisting torsional stresses through shear flow along its cross section wall 3. Longitudinal reinforcement, stirrups and concrete strips between diagonal cracks were considered chords, vertical and diagonal members of truss, respectively
Cao^18^	The thin-walled theory and space truss theory	1. The torsional capacity of the reinforcement was calculated based on the space truss model 2. Concrete internal forces in the X and Y directions were considered balanced with that of stirrups, while Z direction forces were balanced with that of longitudinal reinforcement 3. The tensile force of concrete at the crack was balanced by the shear flow and longitudinal reinforcement tension

### Proposed formula

**Fig. 7:**
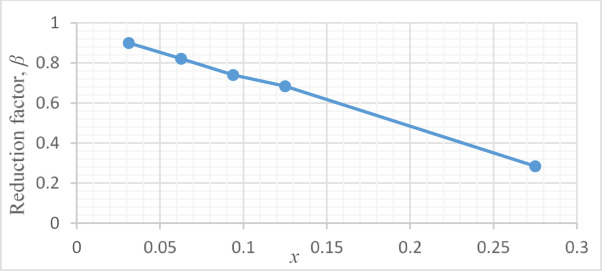
Calculations of the reduction factor $\beta$*.*

The aim of this section is to develop a new formula including all probable influencing parameters to accurately predict the torsional strength of *UHPC* beams particularly, those previously investigated in Section “Numerical results and discussion”–“Introduction”. The proposed new formula incorporates previously mathematically-proven terms with modifications derived from other experimental investigations. Additionally, new parameters, such as web openings, are integrated based on the graphical plots of the *FE* results, Fig. 7. The new model is based on the following assumptions:

1. The torsional resistance of the reinforcement is calculated depending on the space truss model represented in Cao^18^.
2. The torsion load capacity of *UHPC* is calculated accordance with the Chinese code GB 50,010–2010^32^ specifications for torsion in order to account the plastic ultimate torsional strength of *UHPC*.

Obviously, each of the former five formulas consisted of two terms; contributions of concrete ${T}_{1}$ and longitudinal and transverse reinforcing steel ${T}_{2}$. For instance, the first term in Eq. (4)^17^ has proven its potential in expressing the contribution of concrete and fibers to the strength of *UHPC* beams. Here is:

6 $$T_{1} = 0.30\, f_{ut } \,V_{f } \,\lambda_{f} \,W$$

The constant value of 0.30 was adopted in previous studies based on regression analysis. However, as shown in Table 6, this value may underestimate the contribution of concrete and steel fibers. Therefore, a higher constant value is suggested to better align with the experimental results. A comparative analysis was conducted using different values of this constant, as presented in Table 9, which illustrates the correlation between theoretical predictions and experimental results for *UHPC* beams. Among the tested values, a constant of 0.45 yielded the best agreement, with an average experimental-to-theoretical ratio with acceptable coefficient of variation. Accordingly, ${T}_{1}$ in Eq. (6) becomes:

7 $$T_{1} = 0.45 \, f_{ut } \,V_{f } \,\lambda_{f} \, W$$

**Table 9:** Application of Eq. (15) to previous models using different values of the constant.

Study		0.3	0.45	0.50	0.6
Zhou et al.^34^	Mean	0.84	1.01	1.07	1.18
	COV	0.10	0.10	0.09	0.10
Li et al.^35^	Mean	0.81	1.02	1.1	1.23
	COV	0.123	0.117	0.13	0.16
Lina et al.^36^	Mean	0.79	1.02	1.09	1.25
	COV	0.13	0.123	0.129	0.134

The second term in Eq. (5)^18^ can be considered extensive and accurate in expressing the contribution of longitudinal and transverse reinforcing steel:

8 $$T_{2} = 2 \sqrt \zeta \frac{{A_{cor} \,f_{yv } \,A_{sv1} }}{s}$$

But, to ease the calculations and enhance the preciseness of the predicted results, the perimeter of one stirrup ${U}_{st}$ is preferred to be used instead of the interference of shear flow ${U}_{cor}.$ Thus, $\zeta = \frac{S {A}_{sl} {f}_{yl}}{{f}_{yv} {A}_{sv1} {U}_{st}}$.

The contribution of the inclination angle of cracks $\theta$*,* as previously recommended^15,16,18,30^, can be introduced to the proposed new formula as follows:

9 $$T_{u} = \left( {T_{1} + { }T_{2} } \right)\cot \,\theta$$

Thus,

10 $$T_{u} = \left( {0.45{ }\,f_{{ut{ }}} \,V_{{f{ }}} \,\lambda_{f} { }\,W + { }2{ }\sqrt \zeta { }\frac{{A_{cor} { }f_{{yv{ }}} A_{sv1} }}{s}} \right)\cot \,\theta { }$$

According to Kwahk et al.^16^, the recommended value of $\theta$ is 45 degrees. However, for more accurate predictions, the Japanese design guidelines for *UHPC*^33^ and Kwahk et al.^16^ adopted an additional deviation for $\theta$ of 5 degrees and 4.7 degrees, respectively. Furthermore, based on the results of applying Eq. (5)^18^ to the *FE* models, Table 6, any increase in $\theta$ value must be accompanied by a decrease in $\zeta$ value and vice versa. This was also demonstrated by Yang et al.^11^, where $\theta$ depends on the transverse and longitudinal reinforcing steel ratios*.* Accordingly, in this study, $\theta$ is considered 45 degrees and can be changed gradually up to ± 4 degrees depending on $\zeta$ value as follows:

$$\begin{array}{*{20}l} {\theta = 43\,{\text{degrees}}\,{\text{if}}} \hfill & {1.1 \le \zeta \le 1.6} \hfill \\ {\theta = 41\,{\text{degrees}}\,{\text{if}}} \hfill & {\zeta \ge 1.60,} \hfill \\ {\theta = 47\,{\text{degrees}}\,{\text{if}}} \hfill & {\zeta \le 0.70.} \hfill \\ \end{array}$$

The effect of compression pre-force on *θ* is considered as Kwahk et al.^16^ recommended, for *UHPC* prestressed beams, as follows:

11 $$\theta = 0.5{ }\,tan^{ - 1} \left( {\frac{{2\tau_{cr} }}{\delta }} \right){ }$$

where $\delta$ is the compressive stress induced by the prestressing force and ${\tau }_{cr}$ is the shear stress at which inclined cracks initiate at one side of the studied beam under cracking torque and can be calculated as follows:

12 $$\tau_{{cr{ } = { }}} f_{t} \sqrt {1 + \frac{\delta }{{f_{t} }}}$$

The next object was to incorporate, into the derived formula, the contribution of web openings that reduces the torsional strength of *UHPC* beams by the factor $\beta$. The numerical results of the *FE* models in Table 6, supported by the results of a previous study^27^, were graphically plotted in Fig. 7 to represent a mathematical relationship between the reduction factor $\beta$ and the introduced variable $x$, as follows:

13 $$\beta { } = { }0.98{ } - { }2.54{ }\,x{ }$$

where

14 $$x = { }n\frac{{l{ } \times h}}{L \times H}\,{\text{or}}\,x = { }n\frac{{D^{2} }}{L \times H}$$

in which $l$ and $h$ are the length and height of one web opening, respectively and $L$ and $H$ are the length and height of the studied beam, respectively. The term $n$ represents the number of web openings and $D$ is the diameter of openings in case of circular openings.

The term $\beta$ was integrated into the developed new formula is:

15 $$T_{u} = \left( {0.45{ }\,f_{{ut{ }}} \,V_{{f{ }}} \,\lambda_{f} { }\,W + { }2{ }\sqrt \zeta { }\frac{{A_{cor} { }\,f_{{yv{ }}} \,A_{sv1} }}{s}} \right){ }\beta \,\cot \,\theta { }$$

Eventually, the proposed formula, Eq. (15), can be used to determine the torsional strength of *UHPC* beams including concrete tensile strength, fibers properties (diameter, length, and volume fraction), section type (hollow or solid), web openings, compression pre-force, longitudinal steel (yield stress, ratio and diameter) and stirrups (spacing, yield stress and diameter).

#### Validity of proposed formula

**Fig. 8:**
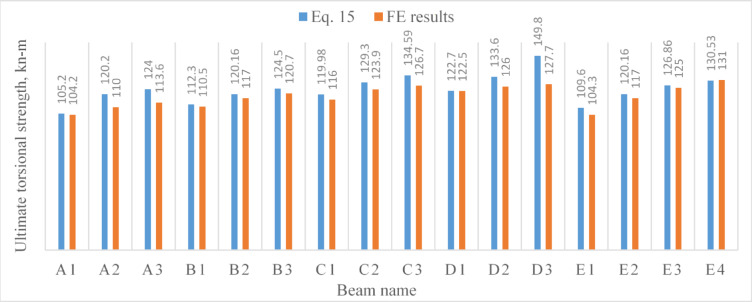
A comparison between the results obtained from the proposed formula and those obtained using Abaqus software for non-precompressed beams.

**Fig. 9:**
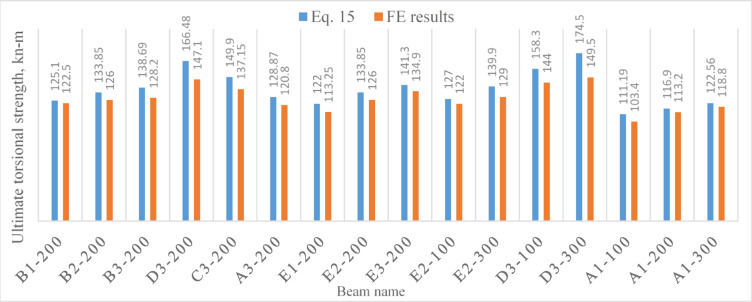
A comparison between the results obtained from the proposed formula and those obtained using Abaqus software for precompressed beams.

**Fig. 10:**
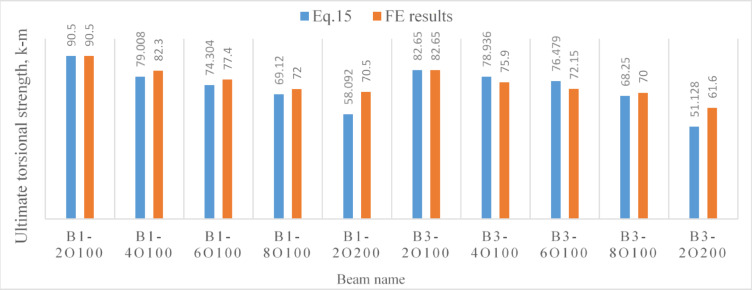
A comparison between the results obtained from the proposed formula and those obtained using Abaqus software for beams with openings.

The credibility of the proposed formula, Eq. (15), was verified by utilizing the results of the 44 *FE* models; ${V}_{f}=2\%$ and ${\lambda }_{f}=100$ (${\lambda }_{f}$ was suggested 65, 90, 91.5, and 113.3 in previous studies^11,15,16,18^ respectively). The predictions of Eq. (15), ${T}_{u}^{Eq.}$, were compared to the *FE* results, ${T}_{u}^{FE}$, and the comparisons were summed in Figs. 8, 9 and 10.

Generally, as Tables 9, 10 and 11 show, the predicted results based on Eq. (15), ${T}_{u}^{Eq.},$ demonstrated an acceptable agreement with those numerically obtained based on *FE* modeling, ${T}_{u}^{FE}.$ The mean values of the ratio $\frac{{T}_{u}^{Eq.}}{{T}_{u}^{FE}}$ was: 1.04, 1.07 and 0.96 for non precompressed beams, precompressed beams and beams with web openings, respectively.

**Table 10:** Results comparison^34^.

Beam	*T*_*u*_^34^, kN-m	${T}_{u}^{Eq.},$ kN-m	$\frac{{T}_{u}^{Eq}}{{T}_{u}}$^34^
HB200T50-1	19.55	21.58	1.10
HB200T50-2	21.12	21.58	1.02
HB300T50-1	44.42	51.7	1.16
HB300T50-2	46.63	51.7	1.11
HB300T80-1	64.11	59.18	0.92
HB300T80-2	61.47	59.18	0.96
CHB300T50-1	62.27	57.35	0.92
CHB300T50-2	63.36	57.35	0.90
Mean %			1.01
COV			0.1

**Table 11:** Results comparison^35^*.*

Beam	*T*_*u*_^35^, kN-m	${T}_{u}^{Eq.},$ kN-m	$\frac{{T}_{u}^{Eq}}{{T}_{u}}$^35^
TL78S36F25	29.04	27.32	0.94
TL78S54F25	28.34	29.12	1.03
TL154S36F25	30.98	30.57	0.986
TL154S54F25	27.83	33.12	1.19
TL78S77F15	26.57	22.52	0.847
TL78S129F15	29.86	25.97	0.87
TL78S77F25	31.98	31.06	0.97
TL78S129F25	36.3	34.51	0.95
TL78S77F35	39	47.95	1.23
TL78S129F35	43.52	51.39	1.18
Mean %			1.02
COV			0.13

For further verification, the proposed equation was calibrated utilizing the results of *UHPC* beams, previously tested in 7 recent studies;^15,16,18,27,34–36^. The predicted torsional strength, ${T}_{u}^{Eq.}$, was then compared to that obtained previously, ${T}_{u}$, Tables 10, 11, 12, 13, 14, 15 and 16. The comparison showed acceptable agreement between the results.

**Table 12:** Results comparison^36^.

Beam	*T*_*u*_^36^, kN-m	${T}_{u}^{Eq.},$ kN-m	$\frac{{T}_{u}^{Eq}}{{T}_{u}}$^36^
B-UHPC	22.9	20.21	0.88
B-UHPC-75	20.6	18.59	0.90
B-UHPC-100	15.7	17.66	1.125
B-UHPC-75-WO	18.9	18.59	0.98
B-UHPC-100-WO	14.8	17.66	1.19
Mean %			1.02
COV			0.12

**Table 13:** Results comparison^27^.

Beam	Tu^27^, kN-m	${T}_{u}^{Eq.},$ kN-m	$\frac{{T}_{u}^{Eq}}{{T}_{u}}$^27^
H	697	613.50	0.881
H100	613	555.90	0.907
H200	448	413	0.922
H300	170	174.85	1.02
Mean			0.934
COV			0.0567

**Table 14:** Results comparison^16^.

Beam	Tu^16^, kN-m	${T}_{u}^{Eq.},$ kN-m	$\frac{{T}_{u}^{Eq}}{{T}_{u}}$^6^
SH-P0-F1.5-L1-S1(D13)	98.65	103.45	1.04
SH-P0-F1-L1-S1	80.65	64.60	0.80
SH-P0-F1.5-L1-S2	101.10	84.50	0.83
SH-P0-F1.5-L1-S1(D10)	95.90	90.15	0.94
SH-P2-F1.5-L1-S1	189.25	191.25	1.01
SH-P4-F1.5-L1-S1	192	191.78	0.999
SH-P4-F1.5-L1-S1-1	145.80	155.75	1.06
SH-P4-F1-L1-S1	190.15	196.37	1.03
Mean %			0.967
COV			0.095

**Table 15:** Results comparison^18^.

Beam	*T*_*u*_^18^, kN-m	${T}_{u}^{Eq.},$ kN-m	$\frac{{T}_{u}^{Eq}}{{T}_{u}}$^18^
L121S126F075	19.49	16.45	0.845
L164S126F075	21.44	19.81	0.924
L214S126F075	23.00	23.80	1.03
L121S084F075	16.50	14.60	0.885
L121S063F075	14.15	13.90	0.982
L121S084F150	21.90	19.90	0.908
L121S084F300	22.33	22.30	0.998
Mean		0.934	
COV		0.0665

**Table 16:** Results comparison^15^.

Beam	Tu^15^, kN-m	${T}_{u}^{Eq.},$ kN-m	$\frac{{T}_{u}^{Eq}}{{T}_{u}}$^15^
UL(1.4)T(1.96)F1(0.5)	26.72	22.65	0.848
UL(2.48)T(1.96)F1(0.5)	31.20	29.53	0.946
UL(2.48)T(2.94)F1(0.5)	32	35.88	1.12
UL(1.4)T(1.96)F1(0.5)H45	25.54	22.52	0.882
Mean			0.949
COV			0.110

#### Applicability and limitations of the model

1. The model is devoted to calculate torsional strength of *UHPC* beams subjected to torsional stresses.
2. The model is applicable only to cross-sections having constant width and height (square or rectangular).
3. To ensure accurate results, UHPC beams should incorporate steel fibers with a volume fraction of 2.5% or less, along with both longitudinal and transverse reinforcement.

## Conclusions

The contribution of the current research can be summarized in the following points:

1. The current research provided comparative study of 5 recent formulas capable of predicting the torsional strength of *UHPC* beams and clarified their deficits to develop a new formula.
2. The developed model incorporates both commonly used parameters and others that have been rarely considered. These parameters include: concrete tensile strength; fiber properties (volume fraction and dimensions); longitudinal reinforcement (ratio and yield strength); transverse reinforcement (ratio, yield strength, and spacing); section type (solid or hollow); web openings (type, number, and dimensions); and compression pre-force, where applicable.
3. The proposed formula demonstrated high accuracy in predicting torsional strength of 44 *FE* models, as well as 46 *UHPC* beams tested in recent studies.
4. Compression pre-force of 10 kN, 20 kN and 30 kN can, based on *UHPC* beam characteristics, enhance its torsional strength by 13%, 15% and 17%, respectively.
5. The increase in the torsional strength of *UHPC* beams, upon applying compression pre-force, was observed to be in direct proportional with the increase in the transverse reinforcing steel content, but in inverse proportional with the increase in the longitudinal reinforcing steel content.
6. Creating two squared web openings, with a side length of 100 mm, in an *UHPC* beam, reduced its torsional strength by only 10%, if the *UHPC* beam was adequately reinforced.
7. Increasing the number of 100mm squared web openings to 4, 6 and 8 reduced the torsional strength of *UHPC* beams by average reduction of 17.5%, 26.5% and 31.5%, respectively.
8. The greater the thickness (up to 0.40 of beam width and 0.30 of its height) of the studied *UHPC* beam, the higher the torsional strength. After that, the improvement in torsional strength was gradually disappeared.
9. The vertical displacement observed in *UHPC* beams without openings at the ultimate load exceeded ten times its corresponding value at the onset of loading.

## Supplementary Information

Supplementary Information.

## Data Availability

The data that support the findings of this study are available from the corresponding author upon reasonable request.
