# Supplementary material for: Effect of compression pre-force and web openings on torsional strength of UHPC hollow beams using numerical and mathematical modeling
Source: Sci Rep. 2025 Jul 16;15:25880. doi: 10.1038/s41598-025-10834-0 (PMC12267853; doi:10.1038/s41598-025-10834-0)
Supplement: Supplementary file 1 — Supplementary Information. [file 41598_2025_10834_MOESM1_ESM.docx]

| 1. Part | Defined in Abaqus software as: |
| --- | --- |
| Concrete | Solid – deformable. |
| Reinforcing steel | Wire – deformable. |
| Torque lever arm | Solid – deformable (with high modulus of elasticity). |
| 1. Material | |
| Concrete | Concrete damaged plasticity (CDP). |
| Reinforcing steel | Wire. |
| Torque lever arm | Steel. |
| 1. Section type | |
| Concrete | Solid – homogenous. |
| Reinforcing steel | Truss. |
| Torque lever arm | Solid – homogenous. |
| 1. Interactions (Constraints) | |
| Between concrete and rigid plate. | Constrains – Tie. |
| Between concrete and reinforcing steel. | Concrete – host region.  Reinforcement – embedded region. |
| Torque lever arm | Rigid body. |
| 1. Meshing | |
| Concrete | Mesh size = 60mm – element shape is (TET). |
| Reinforcing steel | Mesh size = 60mm – element shape is (Truss). |
| Lever arm | Mesh size = 60mm – element shape is (TET). |
| 1. Loading steps | |
| Initial step: for boundary conditions (Fixed-roller).  Step 1: (Static General) for compression pre-force (if any).  Step 2: (Static Riks) for vertical force located at the end of lever arm. | |
| 1. Load Increments | |
| Initial =0.10  Minimum = 0.0001  Maximum = 1.0 | |
